# Economic burden, mortality, and incidence of hospital admissions for cerebrovascular diseases in Brazil from 2017 to 2022: a perspective of the Brazilian Unified Health System

**DOI:** 10.11606/s1518-8787.2025059006781

**Published:** 2025-12-08

**Authors:** Gabriel Medeiros Correia da Silva, Luana Kitagawa Cunha Soares, Ana Clara Ramon Giannelli, Fernando Rocha Oliveira, Luiz Vinicius de Alcantara Sousa, Laércio da Silva Paiva

**Affiliations:** I Centro Universitário Faculdade de Medicina do ABC. Departamento de Saúde da Coletividade. Laboratório de Epidemiologia e Análise de Dados. Santo André, SP, Brasil; II Escola Superior de Ciências da Santa Casa de Misericórdia de Vitória. Programa de Pós-Graduação Stricto Sensu Políticas Públicas e Desenvolvimento Local, Vitória, ES, Brasil

**Keywords:** Cerebrovascular Disorders, Stroke, Hospitalization, Public Expenditures on Health, COVID-19, Mortality, Epidemiology

## Abstract

**OBJECTIVE:**

To analyze hospitalizations, mortality, and costs related to cerebrovascular diseases in Brazil from 2017 to 2022 and to evaluate the impact of the COVID-19 pandemic on these numbers.

**METHODS:**

Data were collected from the Brazilian Unified Health System Information Technology Department via the Hospital and Mortality Information Systems. The 2010 censuses and the inter-census projections (2017 to 2022) of the Brazilian Institute of Geography and Statistics were searched for population data. The mortality rates and incidence of hospital admissions for cerebrovascular diseases were calculated by dividing the number of deaths and the number of hospital admissions due to cerebrovascular diseases by the total population at risk, multiplied by 100,000 inhabitants, respectively. The results were stratified by age groups, regions of Brazil, sex, and calendar years. Rates were standardized by age, following the direct method from the World Health Organization.

**RESULTS:**

Data on mortality from cerebrovascular diseases showed a significant reduction throughout Brazil in general and by sex. The Brazilian Northeast showed a decrease in overall mortality, especially in women, whereas its South showed stationary numbers. Hospitalization rates remain stable, with notable increases in the age groups for younger (from zero to four years of age) and female (from five to nine years of age). Hospitalization costs increased significantly throughout Brazil, with the highest increase in its Northeast, North, Southeast, and Midwest.

**CONCLUSION:**

Mortality rates from cerebrovascular diseases decreased in Brazil, showing regional and age group variations and a worrying increase in young men. Despite stable hospitalizations, hospital costs significantly increased, indicating greater complexity of cases and reinforcing the need for more effective prevention and control strategies.

## INTRODUCTION

The term “stroke” encompasses many subtypes of brain diseases, especially ischemic and hemorrhagic ones^1^. This condition and chronic diseases such as heart disease, diabetes, cancers, cause more than 70% of deaths globally^2^. According to Rochmah et al.^3^, Brazil is among the upper-middle-income countries showing economic losses regarding strokes, alongside Lebanon, Türkiye, South Africa, China, and Colombia. That study also stated that hospitalization length caused the most costs in the analyzed countries. From 2017 to 2020, São Paulo, Brazil, witnessed 40,867 hospitalizations for stroke with no significant changes in hospitalizations from January to June of 2017 and 2019, showing decreases in absolute numbers and average monthly hospitalizations from January to June 2020^4^.

A 2018 study in Brazil estimated that the Brazilian public system spends an average of US$ 305.18 (SD = 185.46) on direct expenses and US$ 2,456.80 (SD = 2,945.20) on indirect expenses per outpatient attendee who was affected by a stroke^5^.

Due to decreased productivity and non-pharmaceutical interventions, the covid-19 pandemic directly caused GDP losses in several countries according to Richards et al.^6^

Cerebrovascular diseases significantly impact health care costs for health systems and the affected individuals. In addition to increasing complications from cardiovascular diseases, including cerebrovascular ones, covid-19 also reduced the demand for medical services due to the fear of contamination, delaying these patients’ diagnosis and treatment. Thus, understanding and quantifying these costs is essential for efficiently allocating resources and developing appropriate health policies^7^.

Thus, this study aims to analyze the costs of hospitalizations for stroke from 2017 to 2022 to assess the effect of the pandemic on these numbers considering its importance for the Brazilian economic and health scenario due to the condition’s high mortality and treatment expenses.

## METHODS

Data regarding the Brazilian population in its five geopolitical macroregions (North, Northeast, Southeast, South, and Midwest) from 2017 to 2022 was analyzed in this time-series ecological study (i.e., an evaluation of population-related factors over time in the same location).

The used data are available on the website of the Brazilian Unified Health System (SUS) Department of Informatics (DATASUS) via its Hospital Information System (SIH/SUS) (which aims to assess information on hospitalizations and hospital costs within SUS) and on the Mortality Information System (which has data on mortality in Brazil, considering annual records from January to December^8^). All available hospital admissions for cerebrovascular diseases following codes I60 to I69 in the 10th revision of the International Classification of Diseases were collected, namely:

- I60: Subarachnoid hemorrhage;
- I61: Intracerebral hemorrhage;
- I62: Other nontraumatic intracranial hemorrhage;
- I63: Cerebral infarction;
- I64: Stroke, not specified as hemorrhagic or ischemic;
- I65: Occlusion and stenosis of precerebral arteries, not resulting in cerebral infarction
- I66: Occlusion and stenosis of cerebral arteries, not resulting in cerebral infarction
- I67: Other cerebrovascular diseases.

This study used deaths, costs, and hospital admissions as variables by sex, age group (from zero to 80 years or older), cerebrovascular disease classifications, regions of Brazil, and year. Collection was carried out on the DATASUS platform^a^.

The 2010 census and the inter-census projections (2017 to 2022) of the Brazilian Institute of Geography and Statistics (which are also available in the DATASUS system) were used to collect data on the overall population.

Based on such information, the rates of mortality due to cerebrovascular diseases were calculated by dividing the number of deaths by these diseases by the total population at risk, multiplied by 100,000 inhabitants. Incidence rates were similarly calculated by considering the number of hospital admissions for cerebrovascular diseases. Data were stratified by age groups, Brazilian regions, sex, and calendar years. Then, mortality rates and hospital admissions were standardized by age, following the direct method proposed by the World Health Organization^9^ (adjustment according to the world standard population).

The collected cost data were stratified by age groups, Brazilian regions, gender, and calendar years and adjusted based on the corresponding Broad Consumer Price Index for each year.

The time-series analysis was conducted using the Prais-Winsten linear regression method, as per Antunes et al.^10^ This method is appropriate when the data show serial autocorrelations due to temporal dependence (which often occurs in population data measures).

In the regression analysis, models were developed in which mortality and hospital admission rates and cerebrovascular disease costs were chosen as dependent variables and calendar year (corresponding to the records of mortality, hospital admissions, and costs) was chosen as the independent variable. The β₁ coefficient of the slope of the straight line, 95% confidence intervals (95%CI), and p-values were estimated to evaluate statistical significance.

This analysis was performed on Stata^®^, version 18.0. Annual percentage variation (APV) and their respective 95%CI was calculated using the following equations: APV = (1 + 10 * b1) * 100% and 95%CI (-1 + 10 * b1min) * 100%; (-1 + 10 * b1max) * 100%. An upward trend in mortality rates, incidence of hospital admissions, and costs was considered if the regression coefficient were positive, as were a downward trend if the regression coefficient were negative. A stationary trend was considered if the probability value (p-value) equaled p > 0.05.

Data were analyzed on the “Data Analysis and Statistical Software for Professionals (Stata^®^),” version 14.0.

Since this research used freely and unrestrictedly accessible secondary data, this study dispensed with assessments by the Human Research Ethics Committee according to Resolution No. 510/2016 of the Brazilian Ministry of Health.

## RESULTS

**Figure 1:**
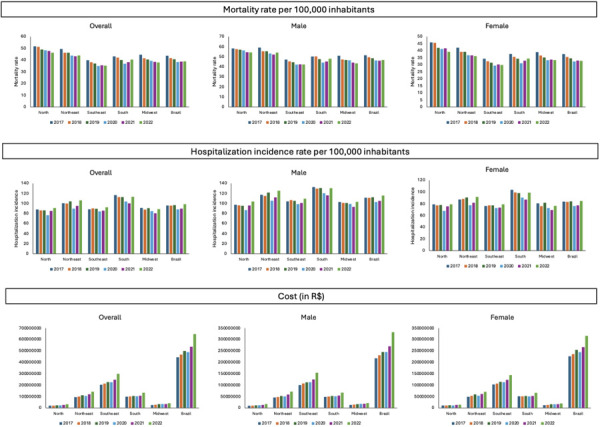
Distribution of mortality rates and the incidence of hospitalization for cerebrovascular diseases according to sex and region of Brazil (2017–2022).

Source: *Sistema de Informação sobre Mortalidade* (SIM) and *Sistema de Informações Hospitalares do Sistema Único de Saúde* (SIH/SUS).

**Figure 2:**
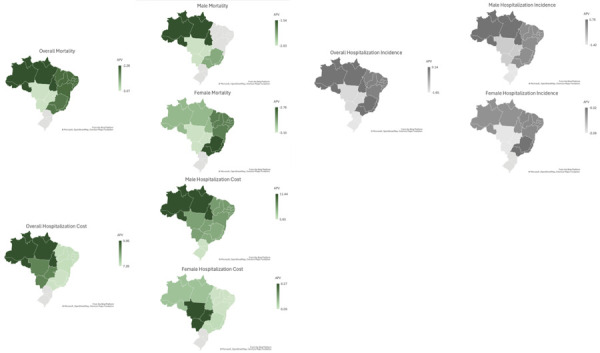
Temporal trends in mortality, incidence, and cost of hospital admissions for cerebrovascular diseases in Brazil from 2017 to 2022, stratified by sex and region. APV: annual percentage variation.

Note: positive values indicate increases whereas negative ones, decreases. Regions in green represent statistically significant differences, whereas regions in gray, stationary variations.

Source: *Sistema de Informação sobre Mortalidade* (SIM) and *Sistema de Informações Hospitalares do Sistema Único de Saúde* (SIH/SUS).

Data on mortality from cerebrovascular diseases show statistically significant reductions in Brazil overall (APV = -2.36; 95%CI: -4.20 to -0.49) and when separately considering men (APV = -2.06; 95%CI: -3.67 to -0.42) and women (APV = -2.73; 95%CI: -4.79 to -0.62). The Brazilian North, Southeast, and Midwest showed a significant reduction in overall mortality rates and for both sexes, with the greatest decreasing variation in the Midwest in overall (APV = -3.07; 95%CI: -4.43 to -1.69), male (APV = -2.83; 95%CI: -3.79 to -1.85), and female mortality rates (APV = -3.10; 95%CI: -5.25 to -0.90). In turn, the Brazilian Northeast showed a decreasing overall mortality rate (APV = -2.39; 95%CI: -3.88 to -0.87) due to a lower female mortality variation (APV = -2.86; 95%CI: -3.95 to -1.76) and a stationary variation in men (APV = -1.88; 95%CI: -3.54 to 0.01). The Brazilian South showed a stationary variation in overall, male, and female mortality rates (Figures 1 and 2).

Data by age groups indicated significant reductions in overall male and female mortality rates in the 60–64 years age group (overall mortality APV = -2.67; 95%CI: -4.05 to -1.27; male APV = -2.42; 95%CI: -4.22 to -0.58; female APV = -3.18; 95%CI: -4.21 to -2.14), 75–79 years age group (overall mortality APV = -3.17; 95%CI: -5.03 to -1.29; male APV = -2.70; 95%CI: -4.44 to -0.92; female APV = -3.74; 95%CI: -5.85 to -1.58), and 80-year or older age group (overall mortality APV = -2.97; 95%CI: -4.90 to -0.99; male APV = -3.04; 95%CI: -4.59 to -1.47; female APV = -2.92; 95%CI: -5.17 to -0.63). Overall mortality, driven by the reduction in male mortality, also showed significant decreases in the 10–14 years age group (overall mortality APV = -6.09; 95%CI: -10.97 to -0.96; male APV = -8.26; 95%CI: -12.55 to -3.76), 50–54 years age group (overall mortality APV = -1.93; 95%CI: -3.45 to -0.38; male APV = -1.92; 95%CI: -2.83 to -1.00), and 65–69 years age group (overall mortality APC = -1.90; 95%CI: -3.68 to -0.08; male APV = -1.65; 95%CI: -2.78 to -0.52), whereas the female rates in these ranges remained stationary. Moreover, overall mortality significant increased due to male mortality in the 25–29 years age group (overall mortality APV = 4.54; 95%CI: 1.07 to 8.13; male APV = 6.41; 95%CI: 1.79 to 11.24), whereas that rate for women remained stable. Significant reductions in female mortality occurred in the 30–34 years age group (APV = -4.41; 95%CI: -7.71 to -0.99), 35–39 years age group (APV = -1.64; 95%CI: -2.80 to -0.46), 45–49 years age group (APV = -1.78; 95%CI: -2.77 to -0.79), 55–59 years age group (APV = -2.42; 95%CI: -4.71 to -0.07), and 70–74 years age group (APV = -2.16; 95%CI: -4.23 to -0.04), whereas the overall and male mortality rates in these age groups remained stationary (Table 1).

**Table 1:** Annual percentage variation in the mortality rate from cerebrovascular diseases in Brazil from 2017 to 2022, stratified by age group and sex.

Overall mortality				
Variables	APV (95%CI)	r^2^	p	Trend
Age group				
0–4 years	0.30 (-14.67 to 17.89)	-	0.962	Stationary
5–9 years	4.78 (-2.26 to 12.32)	0.87	0.136	Stationary
10–14 years	-6.09 (-10.97 to 0.96)	0.88	0.031	Decreasing
15–19 years	-0.97 (-4.27 to 2.44)	0.66	0.468	Stationary
20–24 years	0.69 (-0.91 to 2.32)	0.88	0.298	Stationary
25–29 years	4.54 (1.07 to 8.13)	0.93	0.022	Increasing
30–34 years	-2.02 (-6.31 to 2.46)	0.22	0.273	Stationary
35–39 years	-0.76 (-2.16 to 0.66)	0.99	0.209	Stationary
40–44 years	0.02 (-4.85 to 5.14)	0.95	0.991	Stationary
45–49 years	-1.11 (-2.36 to 0.16)	0.99	0.071	Stationary
50–54 years	-1.93 (-3.45 to 0.38)	0.99	0.026	Decreasing
55–59 years	-1.23 (-3.09 to 0.66)	0.99	0.145	Stationary
60–64 years	-2.67 (-4.05 to 1.27)	0.99	0.006	Decreasing
65–69 years	-1.90 (-3.68 to 0.08)	0.99	0.044	Decreasing
70–74 years	-1.88 (-4.04 to 0.33)	0.99	0.077	Stationary
75–79 years	-3.17 (-5.03 to 1.29)	0.97	0.01	Decreasing
≥ 80 years	-2.97 (-4.90 to 0.99)	0.99	0.014	Decreasing
**Male mortality**				
Age group				
0–4 years	3.69 (-8.87 to 17.98)	0.25	0.48	Stationary
5–9 years	3.52 (-14.02 to 24.63)	0.22	0.633	Stationary
10–14 years	-8.26 (-12.55 to 3.76)	0.88	0.007	Decreasing
15–19 years	-0.47 (-5.03 to 4.32)	0.27	0.795	Stationary
20–24 years	0.47 (-3.75 to 4.87)	-	0.777	Stationary
25–29 years	6.41 (1.79 to 11.24)	0.92	0.018	Increasing
30–34 years	0.00 (-5.69 to 6.05)	0.09	0.998	Stationary
35–39 years	0.46 (-1.98 to 2.95)	0.86	0.633	Stationary
40–44 years	1.04 (-4.76 to 7.19)	-	0.652	Stationary
45–49 years	-0.47 (-2.13 to 1.21)	0.99	0.477	Stationary
50–54 years	-1.92 (-2.83 to 1.00)	0.99	0.005	Decreasing
55–59 years	-0.43 (-1.98 to 1.15)	0.97	0.491	Stationary
60–64 years	-2.42 (-4.22 to 0.58)	0.99	0.022	Decreasing
65–69 years	-1.65 (-2.78 to 0.52)	0.99	0.016	Decreasing
70–74 years	-1.65 (-3.90 to 0.65)	0.99	0.116	Stationary
75–79 years	-2.70 (-4.44 to 0.92)	0.99	0.014	Decreasing
≥ 80 years	-3.04 (-4.59 to 1.47)	0.99	0.006	Decreasing
**Female mortality**				
Age group				
0–4 years	-4.58 (-22.14 to 16.93)	-	0.557	Stationary
59 years	6.41 (-1.94 to 15.46)	0.90	0.102	Stationary
10–14 years	-3.02 (-17.27 to 13.68)	0.49	0.62	Stationary
15–19 years	-1.36 (-4.93 to 2.34)	-	0.36	Stationary
20–24 years	0.21 (-1.55 to 2.00)	0.88	0.763	Stationary
25–29 years	2.18 (-1.02 to 5.49)	0.82	0.133	Stationary
30–34 years	-4.41 (-7.71 to 0.99)	0.90	0.024	Decreasing
35–39 years	-1.64 (-2.80 to 0.46)	0.99	0.018	Decreasing
40–44 years	-0.53 (-4.50 to 3.60)	0.99	0.734	Stationary
45–49 years	-1.78 (-2.77 to 0.79)	0.99	0.008	Decreasing
50–54 years	-2.39 (-4.76 to 0.03)	0.98	0.052	Stationary
55–59 years	-2.42 (-4.71 to 0.07)	0.99	0.046	Decreasing
60–64 years	-3.18 (-4.21 to 2.14)	0.99	0.001	Decreasing
65–69 years	-2.38 (-5.14 to 0.46)	0.99	0.080	Stationary
70–74 years	-2.16 (-4.23 to 0.04)	0.99	0.047	Decreasing
75–79 years	-3.74 (-5.85 to 1.58)	0.98	0.009	Decreasing
≥ 80 years	-2.92 (-5.17 to 0.63)	0.99	0.024	Decreasing

APV: annual percentage variation; 95%CI: 95% confidence interval; r^2^: predictive capacity of the model; p: probability.

Source: *Sistema de Informação sobre Mortalidade* (SIM).

Regarding the incidence of hospital admissions for cerebrovascular diseases, all Brazilian regions showed stationary variations in the incidence rates for overall (APV = -0.34; 95%CI: -3.70 to 3.14), male (APV = -0.09; 95%CI: -3.47 to 3.4), and female hospitalizations (APV = -0.64; 95%CI: -4.00 to 2.84) (Figures 1 and 2).

Moreover, the incidence rates for general (APV = 7.20; 95%CI: 5.53 to 8.90), male (APV = 5.42; 95%CI: 3.60 to 7.27), and female hospitalizations (APV = 10.14; 95%CI: 7.71 to 12.64) significantly increased in the 0–4 age group. The incidence of female hospitalizations showed a significant increase in the 5–9 years age group (APV = 11.20; 95%CI: 4.06 to 18.82), whereas these age groups showed stationary variations in the incidence rates of overall and male hospitalizations (Table 2).

**Table 2:** Annual percentage variation in the incidence rate of hospital admissions for cerebrovascular diseases in Brazil from 2017 to 2022, stratified by age group and sex.

Overall incidence of hospitalizations				
Variables	APV (95%CI)	r^2^	p	Trend
Age group				
0–4 years	7.20 (5.53 to 8.90)	0.99	< 0.001	Increasing
5–9 years	-3.63 (-1.89 to 9.47)	0.48	0.145	Stationary
10–14 years	3.58 (-1.65 to 9.09)	0.76	0.132	Stationary
15–19 years	-0.60 (-5.28 to 4.31)	-	0.747	Stationary
20–24 years	2.24 (-0.80 to 5.37)	0.88	0.111	Stationary
25–29 years	2.93 (-2.51 to 8.67)	0.87	0.214	Stationary
30–34 years	2.07 (-1.21 to 5.46)	0.92	0.156	Stationary
35–39 years	1.08 (-1.89 to 4.14)	0.15	0.375	Stationary
40–44 years	1.72 (-1.53 to 5.08)	0.92	0.218	Stationary
45–49 years	0.27 (-1.35 to 1.91)	0.98	0.669	Stationary
50–54 years	0.36 (-2.63 to 3.44)	0.99	0.758	Stationary
55–59 years	0.32 (-2.31 to 3.03)	0.98	0.753	Stationary
60–64 years	-0.62 (-3.80 to 2.66)	0.88	0.622	Stationary
65–69 years	-0.57 (-3.96 to 2.94)	0.80	0.672	Stationary
70–74 years	-0.44 (-4.19 to 3.46)	0.98	0.766	Stationary
75–79 years	-1.77(-5.95 to 2.61)	0.45	0.32	Stationary
≥ 80 years	-1.31 (-5.31 to 2.86)	0.94	0.427	Stationary
**Male hospitalization incidence**				
Age group				
0–4 years	5.42 (-3.60 to 7.27)	0.98	0.001	Increasing
5–9 years	-1.98 (-6.26 to 2.50)	0.29	0.282	Stationary
10–14 years	3.03 (-1.65 to 7.94)	0.88	0.149	Stationary
15–19 years	-1.49 (-7.06 to 4.42)	0.21	0.515	Stationary
20–24 years	3.29 (-2.24 to 9.14)	0.96	0.178	Stationary
25–29 years	2.93 (-3.07 to 9.29)	0.93	0.253	Stationary
30–34 years	2.96 (-1.59 to 7.72)	0.97	0.148	Stationary
35–39 years	1.87 (-1.12 to 4.96)	0.93	0.16	Stationary
40–44 years	2.74 (-0.91 to 6.53)	0.56	0.107	Stationary
45–49 years	0.77 (-1.30 to 2.88)	0.18	0.363	Stationary
50–54 years	0.78 (-2.14 to 3.79)	0.99	0.504	Stationary
55–59 years	0.70 (-1.73 to 3.18)	0.97	0.474	Stationary
60–64 years	-0.38 (-3.06 to 2.37)	0.93	0.717	Stationary
65–69 years	-0.29 (-3.34 to 2.86)	0.51	0.809	Stationary
70–74 years	-0.03 (-3.92 to 4.01)	0.99	0.983	Stationary
75–79 years	-1.70 (-6.12 to 2.93)	0.15	0.359	Stationary
≥ 80 years	-1.18 (-5.51 to 3.34)	0.96	0.5	Stationary
**Female hospitalization incidence**				
Age group				
0–4 years	10.14 (7.71 to 12.64)	0.99	< 0.001	Increasing
5–9 years	11.20 (4.06 to 18.82)	0.87	0.011	Increasing
10–14 years	4.01 (-3.08 to 11.62)	0.65	0.197	Stationary
15–19 years	0.49 (-3.03 to 4.15)	-	0.72	Stationary
20–24 years	1.01 (-1.82 to 3.93)	0.96	0.381	Stationary
25–29 years	3.05 (-1.85 to 8.21)	0.43	0.162	Stationary
30–34 years	1.19 (-1.23 to 3.67)	0.95	0.246	Stationary
35–39 years	0.38 (-2.88 to 3.74)	0.83	0.768	Stationary
40–44 years	0.84 (-2.08 to 3.83)	0.98	0.475	Stationary
45–49 years	-0.20 (-1.43 to 1.05)	0.99	0.682	Stationary
50–54 years	-0.18 (-3.37 to 3.11)	0.98	0.884	Stationary
55–59 years	-0.20 (-3.11 to 2.79)	0.98	0.857	Stationary
60–64 years	-0.97 (-4.84 to 3.05)	0.80	0.533	Stationary
65–69 years	-0.92 (-4.77 to 3.08)	0.93	0.552	Stationary
70–74 years	-0.89 (-4.49 to 2.85)	0.96	0.539	Stationary
75–79 years	-1.85 (-5.82 to 2.29)	0.62	0.278	Stationary
≥ 80 years	-1.39 (-5.15 to 2.51)	0.88	0.373	Stationary

APV: annual percentage variation; 95%CI: 95% confidence interval; r^2^: predictive capacity of the model; p: probability.

Source: *Sistema de Informações Hospitalares do Sistema Único de Saúde* (SIH/SUS).

Data on the cost of hospitalizations for cerebrovascular diseases showed statistically significant increases overall (APV = 6.95; 95%CI: 2.29 to 11.82) and in men (APV = 7.97; 95%CI: 2.87 to 13.33) and women (APV = 5.92; 95%CI: 1.70 to 10.31). Overall, male, and female cost rates significantly increased in the Brazilian Northeast, North, Southeast, and Midwest, in which the highest variation occurred in the North for overall (APV = 9.06; 95%CI: 3.62 to 14.78) and male hospitalization cost rates (APV = 11.44; 95%CI: 5.16 to 18.09) and in female hospitalization cost rates in the Midwest (APV = 8.27; 95%CI: 4.59 to 12.09). The South showed a stationary variation in the overall and female hospitalization cost rates, with a statistically significant increase only in male hospitalization cost rates (APV = 5.65; 95%CI: 0.39 to 11.18) (Figures 1 and 2).

Data analyzed by age group showed significant increases in the cost rates of overall, male, and female hospitalizations in various age groups. These rates notably increased in the 30–34, 40–49, and 55–80 and over years age groups, varying from 4.66 to 8.95 in the overall hospitalization cost rates (combined with confidence intervals that indicate statistical significance). Specifically, for the 35–39 and 50–54 years age groups, male rates showed a significant increase (APV = 8.10 and 6.27, respectively), influencing the increase in rates in these age groups on the overall cost of hospitalizations, whereas female rates remained stable. In the 15–19 years age group, only female hospitalization cost rates statistically and significantly increased (APV = 3.27; 95%CI: 1.14 to 5.46). A considerable increase occurred in the overall rates in the 20–24 years age group (APV = 3.65; 95%CI: 3.05 to 4.25), although male and female rates, separately analyzed, remained stable in this age group (which can be attributed to the greater statistical power of joint analysis, which captures subtle variations that evade stratification by sex) (Table 3).

**Table 3:** Annual percentage variation in the costs of hospital admissions for cerebrovascular diseases in Brazil from 2017 to 2022, stratified by age group and sex.

Overall hospitalization cost				
Variables	APV (95%CI)	r^2^	p	Trend
Age group				
0–4 years	5.08 (-5.48 to 16.81)	0.77	0.264	Stationary
5–9 years	4.49 (-8.93 to 16.23)	0.84	0.552	Stationary
10–14 years	2.67 (-9.98 to 17.10)	0.91	0.608	Stationary
15–19 years	-1.05 (-6.60 to 4.84)	0.99	0.639	Stationary
20–24 years	3.65 (3.05 to 4.25)	1.00	< 0.001	Increasing
25–29 years	4.61 (-2.04 to 11.71)	0.99	0.129	Stationary
30–34 years	4.66 (3.26 to 6.09)	0.99	0.001	Increasing
35–39 years	5.42 (2.42 to 8.51)	0.86	0.007	Increasing
40–44 years	7.48 (2.98 to 12.17)	0.97	0.009	Increasing
45–49 years	4.84 (2.92 to 6.80)	0.99	0.002	Increasing
50–54 years	4.95 (0.92 to 9.14)	0.99	0.027	Increasing
55–59 years	6.31 (3.42 to 9.28)	0.99	0.004	Increasing
60–64 years	7.05 (2.36 to 11.96)	0.99	0.013	Increasing
65–69 years	7.95 (2.55 to 13.63)	0.99	0.014	Increasing
70–74 years	8.95 (3.09 to 15.14)	0.99	0.013	Increasing
75–79 years	7.21 (0.82 to 14.00)	0.99	0.035	Increasing
≥ 80 years	7.88 (1.68 to 14.47)	0.98	0.024	Increasing
**Male hospitalization cost**				
Age group				
0–4 years	1.99 (-7.85 to 12.89)	0.96	0.618	Stationary
5–9 years	-4.31 (-8.48 to 0.04)	0.99	0.051	Stationary
10–14 years	1.79 (-8.73 to 13.53)	0.88	0.675	Stationary
15–19 years	-3.20 (-11.26 to 5.59)	0.93	0.358	Stationary
20–24 years	3.09 (-0.60 to 6.92)	0.99	0.081	Stationary
25–29 years	5.95 (-1.28 to 13.71)	0.99	0.086	Stationary
30–34 years	4.60 (1.58 to 7.72)	0.92	0.013	Increasing
35–39 years	8.10 (3.15 to 13.28)	0.99	0.010	Increasing
40–44 years	9.65 (3.32 to 16.36)	0.98	0.013	Increasing
45–49 years	6.38 (3.23 to 9.63)	0.99	0.005	Increasing
50–54 years	6.27 (2.28 to 10.41)	0.99	0.012	Increasing
55–59 years	7.50 (4.61 to 10.47)	0.99	0.002	Increasing
60–64 years	7.77 (2.27 to 13.57)	0.99	0.017	Increasing
65–69 years	9.15 (4.29 to 14.24)	0.99	0.006	Increasing
70–74 years	9.47 (3.43 to 15.88)	0.99	0.011	Increasing
75–79 years	7.90 (1.39 to 14.83)	0.99	0.027	Increasing
≥ 80 years	8.55 (1.03 to 16.63)	0.99	0.034	Increasing
**Female hospitalization cost**				
Age group				
0–4 years	9.50 (-4.60 to 25.68)	-	0.141	Stationary
5–9 years	10.99 (-8.65 to 34.87)	0.68	0.211	Stationary
10–14 years	3.73 (-15.02 to 26.62)	0.63	0.716	Stationary
15–19 years	3.27 (1.14 to 5.46)	1.00	0.013	Increasing
20–24 years	4.56 (-1.31 to 10.78)	0.99	0.099	Stationary
25–29 years	3.06 (-3.34 to 9.88)	0.99	0.262	Stationary
30–34 years	5.04 (3.93 to 6.15)	1.00	< 0.001	Increasing
35–39 years	3.36 (-0.61 to 7.49)	0.85	0.079	Stationary
40–44 years	5.73 (2.60 to 8.96)	0.99	0.007	Increasing
45–49 years	3.68 (2.72 to 4.64)	1.00	< 0.001	Increasing
50–54 years	3.44 (-1.32 to 8.42)	0.99	0.117	Stationary
55–59 years	5.07 (2.07 to 8.15)	0.98	0.009	Increasing
60–64 years	6.20 (1.84 to 10.74)	0.96	0.016	Increasing
65–69 years	6.53 (0.52 to 12.91)	0.99	0.039	Increasing
70–74 years	8.31 (2.68 to 14.25)	0.99	0.014	Increasing
75–79 years	6.52 (0.06 to 13.39)	0.98	0.049	Increasing
≥ 80 years	7.35 (2.20 to 12.76)	0.86	0.016	Increasing

APV: annual percentage variation; 95%CI: 95% confidence interval; r^2^: predictive capacity of the model; p: probability.

Source: *Sistema de Informações Hospitalares do Sistema Único de Saúde* (SIH/SUS).

## DISCUSSION

This study found that the greatest reductions in overall mortality and by sex occurred in the Brazilian North, Southeast, and Midwest. This downward trend in mortality rates had occurred from 2008 to 2012 in Brazil, especially in the 15–49 years age group, since all Brazilian regions showed a decline in rates. However, the lowest absolute rates occurred in the South, from 6.32 (95%CI: 6.31 to 6.32) to 5.10 (95%CI: 5.09 to 5.10), whereas the Southeast showed the highest rates, from 8.07 (95%CI: 8.06 to 8.07) to 6.65 (95%CI: 6.64 to 6.65)^11^. Mansur and Favarato^12^ observed the same significant behavior in previous years (1980 to 2012) in the Southeast, South, and Midwest regions but not in the North. These findings resemble the patterns in developed countries, suggesting that access to health services and socioeconomic and cultural factors may justify such trends^13^.

Another Brazilian study showed a reduction in the national mortality rate from cerebrovascular diseases from 1996 to 2015 (APV = -2.4; p = 0.001) as 13 federative units showed significant reductions, including all states in the Midwest (n = 4), Southeast (n = 4), and South (n = 3), encompassing a previous longer period with the same panorama of reduction as that in this study^14^.

André et al.^15^ pointed out that the risk of death from stroke in Brazil drastically decreased from the early 1980s to the early 2000s (from 68.2 to 40.9 per 100,000 inhabitants), a reduction occurring in both sexes and all age groups that exceeded that in developed regions.

This study also indicates that mortality by age group decreased in both sexes in the 60–64 and 75-years-and-older age groups and reductions in women in the 30–39, 45–49, 55–59, and 70–74 years age groups. According to Moreira et al.^16^, the crude mortality rates from stroke from 2000 and 2018 decreased in both sexes in the age groups 25–34, 35–44, 55–64, 65–74, 75–84, and 85 years or older, showing that this scenario of mortality reduction also occurred in a previous period (2000 to 2018) in the age groups this study highlighted from 2017 to 2022.

Lotufo et al.^17^, a previous more comprehensive study, evaluated mortality from cerebrovascular diseases from 1990 to 2015 and showed that the proportion of deaths under 70 years of age decreased by half in the period and that women showed a greater decrease acceleration (evincing that the scenario of mortality reduction had been present since 1990) and a more accentuated reduction.

Analysis showed a stationary incidence of hospitalizations in all regions, except in the 0–4 years age group, which showed an increase in such rates overall and by sex. A study carried out with data from 2009 to 2016 pointed out that the absolute number of hospitalizations for stroke in Brazil increased by 12.1%, from 131,122 in 2009 to 146,950 in 2016, indicating an increase in hospitalizations up to 2016. In that study, age-adjusted hospitalization rate decreased for all age groups from 2009 to 2016^18^, except for younger patients, who showed a 2.1% increase in hospitalizations, whereas this study found a stationary hospitalization from 2017 to 2022 in those aged four years or older. Bernal et al.^19^ point out that, in young adults (aged from 10 to 49 years) in the Brazilian South and Southeast from 2008 to 2018, the incidence rates of hospitalizations due to hemorrhagic strokes decreased and that ischemic strokes showed a stable trend in both sexes, except from 2011 to 2018, evincing an increase in hospitalization rates and signaling that only the incidence of hospitalizations for ischemic stroke from 2008 to 2011 showed a similar scenario of stability to the results in this study (2017 to 2022) for the same age groups and regions.

According to Tanisaka et al.^4^, data for São Paulo, Brazil, from 2017 to 2020 showed no significant changes in stroke hospitalizations from 2017 to 2019, only finding, from January to July 2020, a reduction of 17% in hospitalizations for intracerebral hemorrhages, 32% for cerebral infarctions, 26% for unspecified strokes, and 47% for other cerebrovascular diseases. Thus, such data corroborate the results in this study, except for the results for 2020, if we evaluate São Paulo as an important state in the Brazilian Southeast, the region of this state.

The analysis by age group showed a significant increase in costs in several groups, especially the 30–34, 40–49, and 55–80-years-or-older age groups.

Regarding hospitalization costs, during the studied period and except for the South, which showed a stationary variation in overall costs and for both sexes, all regions (Northeast, North, Southeast, and Midwest) showed an increase in this parameter, which was slightly higher in men. The costs by age group showed an overall increase and in both sexes for the 30–34, 40–44, 45–49, 55–59, 60–64, 65–69, 70–74, 75–79, and 80-year-or-older age groups.

A study conducted in the United States, using patients who suffered acute ischemic strokes and were admitted to services by a national database in 1993–1994 and 2006–2007, evaluated 13 years of data, suggesting that new therapeutic strategies have improved outcomes and increased the cost of hospitalization in adult patients. This resembles the results in this study: increased costs, decreased mortality, and linear hospital admissions^20^.

The literature widely describes modifiable risk factors related to stroke, such as hypertension, diabetes, hyperlipidemia, and smoking, as amenable to interventions, which can contribute to reducing the incidence of stroke and may impact the indicators of mortality, hospitalizations, and hospital costs associated with the disease^21^.

However, the limitations of this study include its lack of distinction of the specific components of hospital costs, which prevents the identification of the factors that directly contributed to the observed increase. Moreover, as this is an ecological study based on aggregated data, extrapolation of findings to individuals requires caution, avoiding direct causal inferences.

## CONCLUSION

The results of this study show a statistically significant downward trend in mortality rates from cerebrovascular diseases in Brazil over the analyzed period overall and according to sex and macroregions, especially on the Brazilian Midwest and Northeast, in addition to the most advanced age groups.

On the other hand, a significant increase in mortality occurred in young men (aged from 25 to 29 years), suggesting the emergence of a new risk profile. In contrast, hospital admission rates have remained broadly stable nationally and regionally. However, the costs related to hospitalizations for cerebrovascular diseases significantly increased in practically all regions, age groups, and genders, indicating an increase in the economic burden of these conditions, even in the face of reduced mortality and stability in hospitalizations.

This scenario may reflect greater attention to the treatment of cerebrovascular diseases by the Brazilian health system, with possible effectiveness in reducing mortality. Future studies should use individual data and include clinical, sociodemographic, and structural variables. This will enable a deeper understanding of the economic determinants associated with stroke and may subsidize more effective public policies.

## Data Availability

The data are freely and unrestricted available via the link: https://datasus.saude.gov.br/

## Footnotes

a Available from: https://datasus.saude.gov.br/informacoes-de-saude-tabnet/, “Informações de Saúde TABNET”, accessing the “Epidemiológicas e Morbidade” section within “Morbidade Hospitalar do SUS (SIH/SUS)”, for macrodata on hospital admissions due to cerebrovascular diseases, and “Estatísticas Vitais” within “Mortalidade desde 1996 pela CID-10”, for macrodata on deaths due to cerebrovascular diseases.
