# Supplementary material for: Economic burden, mortality, and incidence of hospital admissions for cerebrovascular diseases in Brazil from 2017 to 2022: a perspective of the Brazilian Unified Health System
Source: Rev Saude Publica. 2025 Dec 8;59:e44. doi: 10.11606/s1518-8787.2025059006781 (PMC12674689; doi:10.11606/s1518-8787.2025059006781)
Supplement: Translation

# Translation: Carga econômica, mortalidade e incidência de internações hospitalares por doenças cerebrovasculares no Brasil de 2017 a 2022: uma perspectiva do Sistema Único de Saúde

**Authors:** Gabriel Medeiros Correia da Silva, Luana Kitagawa Cunha Soares, Ana Clara Ramon Giannelli, Fernando Rocha Oliveira, Luiz Vinicius de Alcantara Sousa, Laércio da Silva Paiva

**Affiliations:** I Centro Universitário Faculdade de Medicina do ABC. Departamento de Saúde da Coletividade. Laboratório de Epidemiologia e Análise de Dados. Santo André, SP, Brasil; II Escola Superior de Ciências da Santa Casa de Misericórdia de Vitória. Programa de Pós-Graduação Stricto Sensu Políticas Públicas e Desenvolvimento Local, Vitória, ES, Brasil

**Keywords:** Transtornos Cerebrovasculares, Acidente Vascular Cerebral, Hospitalização, Gastos Públicos com Saúde, COVID-19, Mortalidade, Epidemiologia

## Abstract

**OBJETIVO:**

Analisar as hospitalizações, a mortalidade e os custos relacionados às doenças cerebrovasculares no Brasil de 2017 a 2022 e verificar o impacto da pandemia da covid-19 nesses números.

**MÉTODOS:**

Os dados foram coletados junto ao Departamento de Informática do SUS, por meio do Sistema de Informações Hospitalares e pelo Sistema de Informação sobre Mortalidade. Para os dados populacionais foram consultados os censos de 2010 e as projeções intercensitárias (2017 a 2022) do Instituto Brasileiro de Geografia e Estatística. Calcularam-se as taxas de mortalidade e de incidência das admissões hospitalares por doenças cerebrovasculares, por meio da divisão do número de óbitos e do número de admissões hospitalares por doenças cerebrovasculares pela população total em risco, multiplicado por 100.000 habitantes, respectivamente. Os resultados foram estratificados por grupos etários, regiões do Brasil, sexo e anos civis. As taxas foram padronizadas por idade, seguindo o método direto proposto pela Organização Mundial da Saúde.

**RESULTADOS:**

Os dados sobre mortalidade por doenças cerebrovasculares mostraram uma redução significativa em todo o Brasil, tanto no geral quanto por sexo. O Nordeste apresentou uma diminuição da mortalidade geral, principalmente feminina, enquanto a Região Sul manteve números estacionários. As taxas de internação permanecem estáveis, com aumentos notáveis em faixas etárias mais jovens (0 a 4 anos) e femininas (5 a 9 anos). Os custos de internação aumentaram significativamente em todo o país, com maior elevação no Nordeste, Norte, Sudeste e Centro-Oeste.

**CONCLUSÃO:**

Verificou-se redução nas taxas de mortalidade por doenças cerebrovasculares no Brasil, com variações regionais e por faixa etária, embora com aumento preocupante entre homens jovens. Apesar da estabilidade nas internações, os custos hospitalares aumentaram significativamente, indicando maior complexidade dos casos e reforçando a necessidade de estratégias de prevenção e controle mais eficazes.

## INTRODUÇÃO

Acidente vascular cerebral (AVC) é um termo que engloba muitos subtipos de doenças cerebrais, sendo os principais subtipos o AVC isquêmico e hemorrágico^1^. É uma condição que se encontra dentro do grupo de doenças crônicas, assim como cardiopatias, diabetes, cânceres, as quais são responsáveis por mais de 70% das mortes globalmente^2^. Segundo Rochmah et al.^3^, o Brasil se encontra entre os países de renda média alta examinados com perdas econômicas em relação ao AVC, ao lado do Líbano, da Turquia, da África do Sul, da China e da Colômbia. O estudo ainda refere que os países analisados em questão possuem o fator do tempo de permanência internado como o custo dominante. Em conjunto a isso, de 2017 a 2020, houve 40.867 internações por AVC na cidade de São Paulo, Brasil, sem mudanças significativas nas internações entre janeiro e junho dos anos 2017 e 2019, mas com quedas nos números absolutos e internações médias mensais por AVC de janeiro a junho de 2020^4^.

Um estudo realizado em 2018 no Brasil avaliou que o sistema público brasileiro gasta em média US$ 305,18 (DP = 185,46) em gastos diretos e US$ 2.456,80 (DP = 2.945,20) em gastos indiretos por paciente acometido por acidente vascular cerebral em período ambulatorial^5^.

Evidenciando-se o período relacionado à pandemia de covid-19, temos que, devido à diminuição de produtividade e implementação de intervenções não farmacêuticas, a pandemia foi uma causa direta de perdas no PIB em diversos países segundo Richards et al.^6^

As doenças cerebrovasculares têm impacto significativo nos custos de saúde, tanto para os sistemas quanto para os indivíduos acometidos. Além de aumentar as complicações por doenças cardiovasculares, incluindo as condições cerebrovasculares, a infecção causada pela covid-19 também levou à redução na procura por serviços médicos devido ao medo da contaminação, o que resultou em atrasos no diagnóstico e tratamento desses pacientes. Por isso, compreender e quantificar esses custos é essencial para a alocação eficiente de recursos e o desenvolvimento de políticas de saúde adequadas^7^.

Dessa forma, faz-se necessário o presente estudo, a fim de analisar os custos das internações por AVC entre 2017 e 2022 para avaliar o efeito da pandemia nesses números, visto que se trata de uma doença importante, por sua grande mortalidade e gastos para tratamento, para o cenário econômico e da saúde brasileira.

## MÉTODOS

O desenho do estudo compreende a análise de dados referente à população brasileira dentro de suas cinco regiões geopolíticas (Norte, Nordeste, Sudeste, Sul e Centro-Oeste) no período de 2017 a 2022, ou seja, uma análise de fatores relativos a uma população ao longo do tempo num mesmo local, configurando um estudo ecológico de série temporal.

Os dados utilizados estão disponíveis na página eletrônica do Departamento de Informática do SUS (Datasus), por meio do Sistema de Informações Hospitalares do Sistema Único de Saúde (SIH/SUS), que objetiva compreender as informações e realizar o processamento da produção quanto às internações e aos custos hospitalares no âmbito do SUS, e também o Sistema de Informação sobre Mortalidade (SIM), que possui os dados sobre mortalidade no país, considerando as produções anuais de janeiro a dezembro^8^. Além disso, foram coletadas todas as admissões hospitalares por doenças cerebrovasculares, disponíveis na décima revisão do Código Internacional de Doenças (CID-10) nos códigos I60 a I69, sendo eles:

- I60: Hemorragia subaracnoide;
- I61: Hemorragia intracerebral;
- I62: Outras hemorragias intracranianas não-traumáticas;
- I63: Infarto cerebral;
- I64: Acidente vascular cerebral, não especificado como hemorrágico ou isquêmico;
- I65: Oclusão e estenose de artérias pré-cerebrais que não resultam em infarto cerebral;
- I66: Oclusão e estenose de artérias cerebrais que não resultam em infarto cerebral;
- I67: Outras doenças cerebrovasculares.

O estudo utilizou como variáveis óbitos, custos e internações hospitalares por sexo, faixa etária entre 0 e 80 anos ou mais, classificações das doenças cerebrovasculares, regiões do Brasil e ano. A coleta foi realizada pela plataforma do Datasus^a^.

No entanto, para o levantamento dos dados da população total foram utilizados os censos de 2010 e as projeções intercensitárias (2017 a 2022) do Instituto Brasileiro de Geografia e Estatística, que também estão disponíveis no sistema do Datasus.

Com base nas informações disponíveis, calcularam-se as taxas de mortalidade por doenças cerebrovasculares dividindo-se o número de óbitos por essas doenças pela população total em risco, multiplicada por 100.000 habitantes. As taxas de incidência foram calculadas de forma semelhante, considerando o número de admissões hospitalares por doenças cerebrovasculares. Estratificaram-se os dados por grupos etários, regiões do Brasil, sexo e anos civis. Posteriormente, as taxas de mortalidade e de admissões hospitalares foram padronizadas por idade, seguindo o método direto proposto pela Organização Mundial da Saúde^9^ (ajuste de acordo com a população padrão mundial).

Os dados de custos coletados, além de estratificados por grupos etários, regiões brasileiras, sexo e anos civis, foram ajustados com base no Índice de Preços ao Consumidor Amplo correspondente a cada ano.

A análise da série temporal foi conduzida utilizando o método de regressão linear de Prais-Winsten, conforme proposto por Antunes et al.^10^ Esse método é apropriado quando os dados apresentam autocorrelação séria/autocorrelação serial devido à dependência temporal, o que é comum em medidas de dados populacionais.

Na análise de regressão, desenvolveram-se modelos nos quais as variáveis dependentes foram a taxa de mortalidade, a taxa de internações hospitalares e os custos com as doenças cerebrovasculares, e a variável independente foi o ano civil, correspondente aos registros de mortalidade, internações hospitalares e custos. A fim de avaliar a significância estatística, foram obtidos o coeficiente β₁ da inclinação da reta, o intervalo de confiança de 95% (IC95%) e o valor de p.

Essa análise foi realizada utilizando o pacote estatístico Stata^®^ versão 18.0. A variação percentual anual (VPA) foi calculada, juntamente com seus respectivos IC95%, utilizando as seguintes equações: VPA = (1 + 10 * b1) * 100% e IC95% (-1 + 10 * b1min) * 100%; (-1 + 10 * b1max) * 100%. Foi considerada tendência de crescimento das taxas de mortalidade, incidência de admissões hospitalares e dos custos quando o coeficiente de regressão for positivo e tendência de redução quando o coeficiente de regressão for negativo. Considerou-se tendência estacionária quando o valor de probabilidade (p-valor) for p > 0,05.

A análise dos dados foi conduzida por meio do programa “Data Analysis and Statistical Software for Professionals (Stata) version 14.0^®^.

Uma vez que esta pesquisa usou dados secundários, aos quais o acesso é livre e irrestrito, não foi necessária a apreciação do comitê de ética em pesquisa para seres humanos, segundo a Resolução nº 510/2016 do Ministério da Saúde do Brasil.

## RESULTADOS

Os dados de mortalidade por doenças cerebrovasculares revelam reduções estatisticamente significativas no Brasil, tanto de forma geral (VPA = -2,36; IC95% -4,20 a -0,49), quanto ao se considerar separadamente por sexo masculino (VPA = -2,06; IC95% -3,67 a -0,42) e feminino (VPA = -2,73; IC95% -4,79 a -0,62). As regiões Norte, Sudeste e Centro-Oeste mostraram redução significativa nas taxas de mortalidade geral e para ambos os sexos, sendo a maior variação decrescente na região Centro-Oeste nas taxas de mortalidade geral (VPA = -3,07; IC95% -4,43 a -1,69), bem como no sexo masculino (VPA = -2,83; IC95% -3,79 a -1,85) e feminino (VPA = -3,10; IC95% -5,25 a -0,90). A região Nordeste, por sua vez, constatou uma taxa de mortalidade geral decrescente (VPA = -2,39; IC95%: -3,88 a -0,87) às custas da mortalidade feminina (que também apresentou uma taxa decrescente (VPA = -2,86; IC95% -3,95 a -1,76), visto que a variação da taxa de mortalidade masculina mostrou-se estacionária (VPA = -1,88; IC95% -3,54 a 0,01). A região Sul demonstrou uma variação estacionária nas taxas de mortalidade geral, masculina e feminina (Figuras 1 e 2).

**Figura 1:** Distribuição das taxas de mortalidade, incidência de internação por doenças cerebrovasculares, segundo sexo e região do Brasil (2017–2022).

Fonte: Sistema de Informação sobre Mortalidade (SIM) e Sistema de Informações Hospitalares do Sistema Único de Saúde (SIH/SUS).

**Figura 2:** Tendências temporais da mortalidade, incidência e custo das internações hospitalares por doenças cerebrovasculares no Brasil, entre 2017 e 2022, estratificadas por sexo e região. VPA: variação percentual anual.

Nota: valores positivos indicam aumento, valores negativos indicam redução. Regiões em verde representam diferenças estatisticamente significativas, enquanto as regiões em cinza representam as variações estacionárias.

Fonte: Sistema de Informação sobre Mortalidade (SIM) e Sistema de Informações Hospitalares do Sistema Único de Saúde (SIH/SUS).

Os dados por faixa etária indicam reduções significativas nas taxas de mortalidade geral, masculina e feminina nas faixas de 60 a 64 anos (mortalidade geral VPA = -2,67; IC95% -4,05 a -1,27; masculina VPA = -2,42; IC95% -4,22 a -0,58; feminina VPA = -3,18; IC95% -4,21 a -2,14), 75 a 79 anos (mortalidade geral VPA = -3,17; IC95% -5,03 a -1,29; masculina VPA = -2,70; IC95% -4,44 a -0,92; feminina VPA = -3,74; IC95% -5,85 a -1,58) e 80 anos ou mais (mortalidade geral VPA = -2,97; IC95% -4,90 a -0,99; masculina VPA = -3,04; IC95% -4,59 a -1,47; feminina VPA = -2,92; IC95% -5,17 a -0,63). A mortalidade geral, impulsionada pela redução na mortalidade masculina, também mostrou diminuições significativas nas faixas de 10 a 14 anos (mortalidade geral VPA = -6,09; IC95% -10,97 a -0,96; masculina VPA = -8,26; IC95% -12,55 a -3,76), 50 a 54 anos (mortalidade geral VPA = -1,93; IC95% -3,45 a -0,38; masculina VPA = -1,92; IC95% -2,83 a -1,00) e 65 a 69 anos (mortalidade geral VPA = -1,90; IC95% -3,68 a -0,08; masculina VPA = -1,65; IC95% -2,78 a -0,52), enquanto as taxas femininas nessas faixas permaneceram estacionárias. Além disso, houve um aumento significativo na mortalidade geral, devido à mortalidade masculina, na faixa de 25 a 29 anos (mortalidade geral VPA = 4,54; IC95% 1,07 a 8,13; masculina VPA = 6,41; IC95% 1,79 a 11,24), com as taxas femininas mantendo-se estáveis. Reduções significativas na mortalidade feminina foram observadas nas faixas de 30 a 34 anos (VPA = -4,41; IC95% -7,71 a -0,99), 35 a 39 anos (VPA = -1,64; IC95% -2,80 a -0,46), 45 a 49 anos (VPA = -1,78; IC95% -2,77 a -0,79), 55 a 59 anos (VPA = -2,42; IC95% -4,71 a -0,07) e 70 a 74 anos (VPA = -2,16; IC95% -4,23 a -0,04), enquanto as taxas de mortalidade geral e masculina nessas faixas permaneceram estacionárias (Tabela 1).

**Tabela 1:** Variação percentual anual da taxa de mortalidade por doenças cerebrovasculares no Brasil, entre 2017 e 2022, estratificada por faixa etária e sexo.

Mortalidade geral				
Variáveis	VPA (IC95%)	r^2^	p	Tendência
Faixa etária				
0 a 4 anos	0,30 (-14,67 a 17,89)	-	0,962	Estacionária
5 a 9 anos	4,78 (-2,26 a 12,32)	0,87	0,136	Estacionária
10 a 14 anos	-6,09 (-10,97 a -0,96)	0,88	0,031	Decrescente
15 a 19 anos	-0,97 (-4,27 a 2,44)	0,66	0,468	Estacionária
20 a 24 anos	0,69 (-0,91 a 2,32)	0,88	0,298	Estacionária
25 a 29 anos	4,54 (1,07 a 8,13)	0,93	0,022	Crescente
30 a 34 anos	-2,02 (-6,31 a 2,46)	0,22	0,273	Estacionária
35 a 39 anos	-0,76 (-2,16 a 0,66)	0,99	0,209	Estacionária
40 a 44 anos	0,02 (-4,85 a 5,14)	0,95	0,991	Estacionária
45 a 49 anos	-1,11 (-2,36 a 0,16)	0,99	0,071	Estacionária
50 a 54 anos	-1,93 (-3,45 a -0,38)	0,99	0,026	Decrescente
55 a 59 anos	-1,23 (-3,09 a 0,66)	0,99	0,145	Estacionária
60 a 64 anos	-2,67 (-4,05 a -1,27)	0,99	0,006	Decrescente
65 a 69 anos	-1,90 (-3,68 a -0,08)	0,99	0,044	Decrescente
70 a 74 anos	-1,88 (-4,04 a 0,33)	0,99	0,077	Estacionária
75 a 79 anos	-3,17 (-5,03 a -1,29)	0,97	0,01	Decrescente
≥ 80 anos	-2,97 (-4,90 a -0,99)	0,99	0,014	Decrescente
**Mortalidade masculina**				
Faixa etária				
0 a 4 anos	3,69 (-8,87 a 17,98)	0,25	0,48	Estacionária
5 a 9 anos	3,52 (-14,02 a 24,63)	0,22	0,633	Estacionária
10 a 14 anos	-8,26 (-12,55 a -3,76)	0,88	0,007	Decrescente
15 a 19 anos	-0,47 (-5,03 a 4,32)	0,27	0,795	Estacionária
20 a 24 anos	0,47 (-3,75 a 4,87)	-	0,777	Estacionária
25 a 29 anos	6,41 (1,79 a 11,24)	0,92	0,018	Crescente
30 a 34 anos	0,00 (-5,69 a 6,05)	0,09	0,998	Estacionária
35 a 39 anos	0,46 (-1,98 a 2,95)	0,86	0,633	Estacionária
40 a 44 anos	1,04 (-4,76 a 7,19)	-	0,652	Estacionária
45 a 49 anos	-0,47 (-2,13 a 1,21)	0,99	0,477	Estacionária
50 a 54 anos	-1,92 (-2,83 a -1,00)	0,99	0,005	Decrescente
55 a 59 anos	-0,43 (-1,98 a 1,15)	0,97	0,491	Estacionária
60 a 64 anos	-2,42 (-4,22 a -0,58)	0,99	0,022	Decrescente
65 a 69 anos	-1,65 (-2,78 a -0,52)	0,99	0,016	Decrescente
70 a 74 anos	-1,65 (-3,90 a 0,65)	0,99	0,116	Estacionária
75 a 79 anos	-2,70 (-4,44 a -0,92)	0,99	0,014	Decrescente
≥ 80 anos	-3,04 (-4,59 a -1,47)	0,99	0,006	Decrescente
**Mortalidade feminina**				
Faixa etária				
0 a 4 anos	-4,58 (-22,14 a 16,93)	-	0,557	Estacionária
5 a 9 anos	6,41 (-1,94 a 15,46)	0,90	0,102	Estacionária
10 a 14 anos	-3,02 (-17,27 a 13,68)	0,49	0,62	Estacionária
15 a 19 anos	-1,36 (-4,93 a 2,34)	-	0,36	Estacionária
20 a 24 anos	0,21 (-1,55 a 2,00)	0,88	0,763	Estacionária
25 a 29 anos	2,18 (-1,02 a 5,49)	0,82	0,133	Estacionária
30 a 34 anos	-4,41 (-7,71 a -0,99)	0,90	0,024	Decrescente
35 a 39 anos	-1,64 (-2,80 a -0,46)	0,99	0,018	Decrescente
40 a 44 anos	-0,53 (-4,50 a 3,60)	0,99	0,734	Estacionária
45 a 49 anos	-1,78 (-2,77 a -0,79)	0,99	0,008	Decrescente
50 a 54 anos	-2,39 (-4,76 a 0,03)	0,98	0,052	Estacionária
55 a 59 anos	-2,42 (-4,71 a -0,07)	0,99	0,046	Decrescente
60 a 64 anos	-3,18 (-4,21 a -2,14)	0,99	0,001	Decrescente
65 a 69 anos	-2,38 (-5,14 a 0,46)	0,99	0,080	Estacionária
70 a 74 anos	-2,16 (-4,23 a -0,04)	0,99	0,047	Decrescente
75 a 79 anos	-3,74 (-5,85 a -1,58)	0,98	0,009	Decrescente
≥ 80 anos	-2,92 (-5,17 a -0,63)	0,99	0,024	Decrescente

VPA: variação percentual anual; IC95%: intervalo de confiança de 95%; r^2^: capacidade preditiva do modelo; p: probabilidade.

Fonte: Sistema de Informação sobre Mortalidade (SIM).

Em relação à incidência de internações hospitalares por doenças cerebrovasculares, observa-se que todas as regiões brasileiras mostraram variações estacionárias nas taxas de incidência de internações gerais (VPA = -0,34; IC95% -3,70 a 3,14), masculina (VPA = -0,09; IC95% -3,47 a 3,4) e feminina (VPA = -0,64; IC95%: -4,00 a 2,84), assim como no país em sua totalidade (Figuras 1 e 2).

Nos dados por faixa etária, as taxas de incidência por internação geral (VPA = 7,20; IC95% 5,53 a 8,90), masculina (VPA = 5,42; IC95% 3,60 a 7,27) e feminina (VPA = 10,14; IC95% 7,71 a 12,64) mostraram significativo aumento na faixa de 0 a 4 anos. A incidência de internações femininas apresentou significativo aumento na faixa de 5 a 9 anos (VPA = 11,20; IC95% 4,06 a 18,82), enquanto as variações das taxas de incidência de internações geral e masculina nessas faixas demonstraram-se estacionárias (Tabela 2).

**Tabela 2:** Variação percentual anual da taxa de incidência de internações hospitalares por doenças cerebrovasculares no Brasil, entre 2017 e 2022, estratificada por faixa etária e sexo.

Incidência de internações geral				
Variáveis	VPA (IC95%)	r^2^	p	Tendência
Faixa etária				
0 a 4 anos	7,20 (5,53 a 8,90)	0,99	< 0,001	Crescente
5 a 9 anos	-3,63 (-1,89 a 9,47)	0,48	0,145	Estacionária
10 a 14 anos	3,58 (-1,65 a 9,09)	0,76	0,132	Estacionária
15 a 19 anos	-0,60 (-5,28 a 4,31)	-	0,747	Estacionária
20 a 24 anos	2,24 (-0,80 a 5,37)	0,88	0,111	Estacionária
25 a 29 anos	2,93 (-2,51 a 8,67)	0,87	0,214	Estacionária
30 a 34 anos	2,07 (-1,21 a 5,46)	0,92	0,156	Estacionária
35 a 39 anos	1,08 (-1,89 a 4,14)	0,15	0,375	Estacionária
40 a 44 anos	1,72 (-1,53 a 5,08)	0,92	0,218	Estacionária
45 a 49 anos	0,27 (-1,35 a 1,91)	0,98	0,669	Estacionária
50 a 54 anos	0,36 (-2,63 a 3,44)	0,99	0,758	Estacionária
55 a 59 anos	0,32 (-2,31 a 3,03)	0,98	0,753	Estacionária
60 a 64 anos	-0,62 (-3,80 a 2,66)	0,88	0,622	Estacionária
65 a 69 anos	-0,57 (-3,96 a 2,94)	0,80	0,672	Estacionária
70 a 74 anos	-0,44 (-4,19 a 3,46)	0,98	0,766	Estacionária
75 a 79 anos	-1,77(-5,95 a 2,61)	0,45	0,32	Estacionária
≥ 80 anos	-1,31 (-5,31 a 2,86)	0,94	0,427	Estacionária
**Incidência de internações masculina**				
Faixa etária				
0 a 4 anos	5,42 (3,60 a 7,27)	0,98	0,001	Crescente
5 a 9 anos	-1,98 (-6,26 a 2,50)	0,29	0,282	Estacionária
10 a 14 anos	3,03 (-1,65 a 7,94)	0,88	0,149	Estacionária
15 a 19 anos	-1,49 (-7,06 a 4,42)	0,21	0,515	Estacionária
20 a 24 anos	3,29 (-2,24 a 9,14)	0,96	0,178	Estacionária
25 a 29 anos	2,93 (-3,07 a 9,29)	0,93	0,253	Estacionária
30 a 34 anos	2,96 (-1,59 a 7,72)	0,97	0,148	Estacionária
35 a 39 anos	1,87 (-1,12 a 4,96)	0,93	0,16	Estacionária
40 a 44 anos	2,74 (-0,91 a 6,53)	0,56	0,107	Estacionária
45 a 49 anos	0,77 (-1,30 a 2,88)	0,18	0,363	Estacionária
50 a 54 anos	0,78 (-2,14 a 3,79)	0,99	0,504	Estacionária
55 a 59 anos	0,70 (-1,73 a 3,18)	0,97	0,474	Estacionária
60 a 64 anos	-0,38 (-3,06 a 2,37)	0,93	0,717	Estacionária
65 a 69 anos	-0,29 (-3,34 a 2,86)	0,51	0,809	Estacionária
70 a 74 anos	-0,03 (-3,92 a 4,01)	0,99	0,983	Estacionária
75 a 79 anos	-1,70 (-6,12 a 2,93)	0,15	0,359	Estacionária
≥ 80 anos	-1,18 (-5,51 a 3,34)	0,96	0,5	Estacionária
**Incidência de internações feminina**				
Faixa etária				
0 a 4 anos	10,14 (7,71 a 12,64)	0,99	< 0,001	Crescente
5 a 9 anos	11,20 (4,06 a 18,82)	0,87	0,011	Crescente
10 a 14 anos	4,01 (-3,08 a 11,62)	0,65	0,197	Estacionária
15 a 19 anos	0,49 (-3,03 a 4,15)	-	0,72	Estacionária
20 a 24 anos	1,01 (-1,82 a 3,93)	0,96	0,381	Estacionária
25 a 29 anos	3,05 (-1,85 a 8,21)	0,43	0,162	Estacionária
30 a 34 anos	1,19 (-1,23 a 3,67)	0,95	0,246	Estacionária
35 a 39 anos	0,38 (-2,88 a 3,74)	0,83	0,768	Estacionária
40 a 44 anos	0,84 (-2,08 a 3,83)	0,98	0,475	Estacionária
45 a 49 anos	-0,20 (-1,43 a 1,05)	0,99	0,682	Estacionária
50 a 54 anos	-0,18 (-3,37 a 3,11)	0,98	0,884	Estacionária
55 a 59 anos	-0,20 (-3,11 a 2,79)	0,98	0,857	Estacionária
60 a 64 anos	-0,97 (-4,84 a 3,05)	0,80	0,533	Estacionária
65 a 69 anos	-0,92 (-4,77 a 3,08)	0,93	0,552	Estacionária
70 a 74 anos	-0,89 (-4,49 a 2,85)	0,96	0,539	Estacionária
75 a 79 anos	-1,85 (-5,82 a 2,29)	0,62	0,278	Estacionária
≥ 80 anos	-1,39 (-5,15 a 2,51)	0,88	0,373	Estacionária

VPA: variação percentual anual; IC95%: intervalo de confiança de 95%; r^2^: capacidade preditiva do modelo; p: probabilidade.

Fonte: Sistema de Informações Hospitalares do Sistema Único de Saúde (SIH/SUS).

Os dados de custo de internações por doenças cerebrovasculares revelam aumentos estatisticamente significativos no Brasil, tanto de forma geral (VPA = 6,95; IC95% 2,29 a 11,82) quanto ao se considerar separadamente pelo sexo masculino (VPA = 7,97; IC95% 2,87 a 13,33) e feminino (VPA = 5,92; IC95% 1,70 a 10,31). Verifica-se que as taxas de custo geral, masculina e feminina mostraram significativo aumento nas regiões Nordeste, Norte, Sudeste e Centro-Oeste, sendo a maior variação observada na região Norte nas taxas de custo de internações geral (VPA = 9,06; IC95% 3,62 a 14,78) e masculina (VPA = 11,44; IC95% 5,16 a 18,09), e na região Centro-Oeste na taxa de custo de internações feminina (VPA = 8,27; IC95% 4,59 a 12,09). A região Sul, por sua vez, mostrou uma variação estacionária nas taxas de custo de internações geral e feminina, apresentando um aumento estatisticamente significativo apenas na taxa de custo de internações masculina (VPA = 5,65; IC95% 0,39 a 11,18) (Figuras 1 e 2).

Os dados analisados por faixa etária revelam aumentos significativos nas taxas de custo de internações geral, masculina e feminina em várias faixas etárias. Essas taxas mostraram aumentos notáveis nas faixas etárias de 30 a 34 anos, 40 a 49 anos e 55 a 80 anos ou mais, com variações entre 4,66 e 8,95 nas taxas de custo de internações geral, aliadas aos intervalos de confiança que indicam significância estatística. Especificamente, para as faixas de 35 a 39 anos e 50 a 54 anos, o aumento foi significativo nas taxas masculinas (VPA = 8,10 e 6,27, respectivamente), influenciando o aumento das taxas nessas mesmas faixas etárias no custo de internações geral, enquanto as taxas femininas permaneceram estáveis. Na faixa etária de 15 a 19 anos, apenas a taxa de custo de internação feminina apresentou aumento estatisticamente significativo (VPA = 3,27; IC95% 1,14 a 5,46). Um aumento considerável também foi observado nas taxas gerais de 20 a 24 anos (VPA = 3,65; IC95% 3,05 a 4,25), apesar das taxas masculina e feminina, analisadas separadamente, se manterem estáveis nessa faixa etária (esse achado pode ser atribuído ao maior poder estatístico da análise conjunta, que capta variações sutis não detectáveis quando os dados são estratificados por sexo), (Tabela 3).

**Tabela 3:** Variação percentual anual dos custos com internações hospitalares por doenças cerebrovasculares no Brasil, entre 2017 e 2022, estratificada por faixa etária e sexo.

Custo de internações geral				
Variáveis	VPA (IC95%)	r^2^	p	Tendência
Faixa etária				
0 a 4 anos	5,08 (-5,48 a 16,81)	0,77	0,264	Estacionária
5 a 9 anos	4,49 (-8,93 a 16,23)	0,84	0,552	Estacionária
10 a 14 anos	2,67 (-9,98 a 17,10)	0,91	0,608	Estacionária
15 a 19 anos	-1,05 (-6,60 a 4,84)	0,99	0,639	Estacionária
20 a 24 anos	3,65 (3,05 a 4,25)	1,00	< 0,001	Crescente
25 a 29 anos	4,61 (-2,04 a 11,71)	0,99	0,129	Estacionária
30 a 34 anos	4,66 (3,26 a 6,09)	0,99	0,001	Crescente
35 a 39 anos	5,42 (2,42 a 8,51)	0,86	0,007	Crescente
40 a 44 anos	7,48 (2,98 a 12,17)	0,97	0,009	Crescente
45 a 49 anos	4,84 (2,92 a 6,80)	0,99	0,002	Crescente
50 a 54 anos	4,95 (0,92 a 9,14)	0,99	0,027	Crescente
55 a 59 anos	6,31 (3,42 a 9,28)	0,99	0,004	Crescente
60 a 64 anos	7,05 (2,36 a 11,96)	0,99	0,013	Crescente
65 a 69 anos	7,95 (2,55 a 13,63)	0,99	0,014	Crescente
70 a 74 anos	8,95 (3,09 a 15,14)	0,99	0,013	Crescente
75 a 79 anos	7,21 (0,82 a 14,00)	0,99	0,035	Crescente
≥ 80 anos	7,88 (1,68 a 14,47)	0,98	0,024	Crescente
**Custo de internações masculina**				
Faixa etária				
0 a 4 anos	1,99 (-7,85 a 12,89)	0,96	0,618	Estacionária
5 a 9 anos	-4,31 (-8,48 a 0,04)	0,99	0,051	Estacionária
10 a 14 anos	1,79 (-8,73 a 13,53)	0,88	0,675	Estacionária
15 a 19 anos	-3,20 (-11,26 a 5,59)	0,93	0,358	Estacionária
20 a 24 anos	3,09 (-0,60 a 6,92)	0,99	0,081	Estacionária
25 a 29 anos	5,95 (-1,28 a 13,71)	0,99	0,086	Estacionária
30 a 34 anos	4,60 (1,58 a 7,72)	0,92	0,013	Crescente
35 a 39 anos	8,10 (3,15 a 13,28)	0,99	0,010	Crescente
40 a 44 anos	9,65 (3,32 a 16,36)	0,98	0,013	Crescente
45 a 49 anos	6,38 (3,23 a 9,63)	0,99	0,005	Crescente
50 a 54 anos	6,27 (2,28 a 10,41)	0,99	0,012	Crescente
55 a 59 anos	7,50 (4,61 a 10,47)	0,99	0,002	Crescente
60 a 64 anos	7,77 (2,27 a 13,57)	0,99	0,017	Crescente
65 a 69 anos	9,15 (4,29 a 14,24)	0,99	0,006	Crescente
70 a 74 anos	9,47 (3,43 a 15,88)	0,99	0,011	Crescente
75 a 79 anos	7,90 (1,39 a 14,83)	0,99	0,027	Crescente
≥ 80 anos	8,55 (1,03 a 16,63)	0,99	0,034	Crescente
**Custo de internações feminina**				
Faixa etária				
0 a 4 anos	9,50 (-4,60 a 25,68)	-	0,141	Estacionária
5 a 9 anos	10,99 (-8,65 a 34,87)	0,68	0,211	Estacionária
10 a 14 anos	3,73 (-15,02 a 26,62)	0,63	0,716	Estacionária
15 a 19 anos	3,27 (1,14 a 5,46)	1,00	0,013	Crescente
20 a 24 anos	4,56 (-1,31 a 10,78)	0,99	0,099	Estacionária
25 a 29 anos	3,06 (-3,34 a 9,88)	0,99	0,262	Estacionária
30 a 34 anos	5,04 (3,93 a 6,15)	1,00	< 0,001	Crescente
35 a 39 anos	3,36 (-0,61 a 7,49)	0,85	0,079	Estacionária
40 a 44 anos	5,73 (2,60 a 8,96)	0,99	0,007	Crescente
45 a 49 anos	3,68 (2,72 a 4,64)	1,00	< 0,001	Crescente
50 a 54 anos	3,44 (-1,32 a 8,42)	0,99	0,117	Estacionária
55 a 59 anos	5,07 (2,07 a 8,15)	0,98	0,009	Crescente
60 a 64 anos	6,20 (1,84 a 10,74)	0,96	0,016	Crescente
65 a 69 anos	6,53 (0,52 a 12,91)	0,99	0,039	Crescente
70 a 74 anos	8,31 (2,68 a 14,25)	0,99	0,014	Crescente
75 a 79 anos	6,52 (0,06 a 13,39)	0,98	0,049	Crescente
≥ 80 anos	7,35 (2,20 a 12,76)	0,86	0,016	Crescente

VPA: variação percentual anual; IC95%: intervalo de confiança de 95%; r^2^: capacidade preditiva do modelo; p: probabilidade.

Fonte: Sistema de Informações Hospitalares do Sistema Único de Saúde (SIH/SUS).

## DISCUSSÃO

No presente estudo, verificou-se que as maiores reduções na mortalidade geral e por sexo ocorreram nas regiões Norte, Sudeste e Centro-Oeste. Essa tendência de queda nas taxas de mortalidade já havia sido observada no Brasil entre 2008 e 2012, especialmente na faixa etária de 15 a 49 anos, em que todas as regiões brasileiras apresentaram declínio nas taxas. No entanto, as menores taxas absolutas foram registradas na região Sul, variando de 6,32 (IC95% 6,31 a 6,32) a 5,10 (IC95% 5,09 a 5,10), enquanto as maiores taxas ocorreram na região Sudeste, com variação de 8,07 (IC95% 8,06 a 8,07) a 6,65 (IC95% 6,64 a 6,65)^11^. Ademais, um estudo conduzido por Mansur e Favarato^12^ observou o mesmo comportamento significativo em anos anteriores (1980 a 2012), nas regiões Sudeste, Sul e Centro-Oeste, com exceção da região Norte do Brasil. Esses achados se assemelham aos padrões observados em países desenvolvidos, sugerindo que o acesso aos serviços de saúde, juntamente com fatores socioeconômicos e culturais, pode justificar tais tendências^13^.

Outro estudo brasileiro que analisou o período de 1996 a 2015 evidenciou a redução da taxa de mortalidade nacional por doenças cerebrovasculares (APC = -2,4; p = 0,001), com 13 unidades federativas apresentando resultados significativos de redução, incluindo todos os estados das regiões Centro-Oeste (n = 4), Sudeste (n = 4) e Sul (n = 3), abrangendo um período anterior maior com o mesmo panorama de redução do período presente no estudo^14^.

André et al.^15^ apontam que o risco de morte por AVC no Brasil diminui drasticamente entre o início de 1980 e o início de 2000, de 68,2 para 40,9 por 100.000 habitantes, sendo a redução presente em ambos os sexos e todas as faixas etárias e mais acentuada nas regiões desenvolvidas.

Temos também que o presente estudo indica que a mortalidade por faixa etária apresentou reduções em ambos os sexos nas faixas de 60 a 64 anos e 75 anos ou superior, além de apresentar reduções no sexo feminino nas faixas etárias de 30 a 39 anos, 45 a 49 anos, 55 a 59 anos e 70 a 74 anos. De acordo com Moreira et al.^16^, as taxas brutas de mortalidade por acidente vascular cerebral entre 2000 e 2018 tiveram reduções em ambos os sexos nas faixas etárias de 25 a 34 anos, 35 a 44 anos, 55 a 64 anos, 65 a 74 anos, 75 a 84 anos e 85 anos ou superior, mostrando que esse cenário de redução da mortalidade estava caracterizado no período anterior (2000 a 2018) também nas faixas etárias destacadas no presente estudo, no período estudado de 2017 a 2022.

Lotufo et al.^17^ também realizou um estudo anterior mais abrangente, avaliando a mortalidade por doenças cerebrovasculares entre 1990 e 2015, e revelou que a proporção de mortes abaixo dos 70 anos de idade reduziu pela metade no período, sendo que a aceleração da queda foi maior entre as mulheres, mostrando que o cenário de redução de mortalidade já se apresentava desde 1990, além de mostrar que o sexo feminino já vinha apresentando uma redução mais acentuada.

Dando seguimento à análise dos resultados, temos que a incidência de internações se mostraram estacionárias em todas as regiões, exceto na faixa etária de 0 a 4 anos, que apresentou um aumento nas taxas de incidência de internações geral e por sexo. Um estudo realizado com os dados de 2009 a 2016 apontou que o número absoluto de internações por AVC no Brasil aumentou 12,1%, passando de 131.122 em 2009 para 146.950 em 2016, indicando um cenário de aumento nos números de internações até 2016. No mesmo estudo, a taxa de internações ajustada por idade diminuiu para todos os grupos etários de 2009 a 2016^18^, exceto para os pacientes mais jovens, em que houve um aumento de 2,1% das internações, enquanto no presente estudo a taxa de internações mostrou-se estacionária no período de 2017 a 2022 a partir dos 4 anos de idade. Bernal et al.^19^ apontam que, em adultos jovens (10 a 49 anos) nas regiões Sul e Sudeste do Brasil no período de 2008 a 2018, as taxas de incidência de internações revelaram uma redução nos casos de AVC hemorrágico e apresentarem uma tendência estável em relação aos casos de AVC isquêmico em ambos os sexos, exceto no período de 2011 a 2018, no qual apresentaram um aumento das taxas de internações, sinalizando que nesse período apenas a incidência de internações por AVC isquêmico de 2008 a 2011 mostraram um cenário similar de estabilidade aos resultados observados no presente estudo (2017 a 2022) para as mesmas faixas etárias e regiões.

De acordo com Tanisaka et al.⁴, com dados abrangendo períodos de 2017 a 2020 em São Paulo, Brasil, foi avaliado que não houve mudanças significativas nas internações por AVC entre 2017 e 2019, apresentando apenas, no período de janeiro a julho de 2020, uma redução de 17% nos internamentos por hemorragia intracerebral, 32% por infarto cerebral, 26% por acidente vascular cerebral não especificado e 47% por outras doenças cerebrovasculares. Sendo assim, tais dados corroboram os resultados apresentados no presente estudo, contrapondo-se apenas com os resultados referentes ao período de 2020, se avaliarmos São Paulo como um importante estado da região Sudeste, que é a região que abrange o estado neste estudo.

A análise por faixa etária revelou crescimento significativo dos custos em diversas faixas, destacando-se os grupos de 30 a 34, 40 a 49, e de 55 a 80 anos ou mais.

Em relação aos custos de internações, temos que no período do estudo, com exceção da região Sul que apresentou uma variação estacionária nos custos geral e para ambos os sexos, todas as demais regiões apresentaram aumento nesse parâmetro estudado (Nordeste, Norte, Sudeste e Centro-Oeste), sendo esses aumentos ligeiramente maiores no sexo masculino. Os custos por faixa etária exibiram aumento nas faixas etárias geral e em ambos os sexos de 30 a 34 anos, 40 a 44 anos, 45 a 49 anos, 55 a 59 anos, 60 a 64 anos, 65 a 69 anos, 70 a 74 anos, 75 a 79 anos e 80 anos ou mais.

Colocando em comparação com análises feitas em outros países, temos que um estudo realizado nos Estados Unidos utilizando o número de pacientes com AVC isquêmico agudo admitidos em serviços por um banco de dados nacional em 1993–1994 e 2006–2007, evidenciando um período de 13 anos de análise, sugere que novas estratégias terapêuticas melhoraram os resultados e aumentaram o custo da hospitalização entre pacientes adultos, o que se assemelha aos resultados do presente estudo, com aumento dos custos, diminuição da mortalidade e linearidade das internações hospitalares brasileiras^20^.

Os fatores de risco modificáveis relacionados ao AVC, como hipertensão, diabetes, hiperlipidemia e tabagismo, são amplamente reconhecidos na literatura como passíveis de intervenção, o que pode contribuir para a redução da incidência de AVC e, potencialmente, impactar os indicadores de mortalidade, internações e custos hospitalares associados à doença^21^.

No entanto, o presente estudo apresenta limitações quanto à ausência de discriminação dos componentes específicos dos custos hospitalares, o que impede a identificação dos fatores que contribuíram diretamente para o aumento observado. Além disso, por se tratar de um estudo ecológico, baseado em dados agregados, a extrapolação dos achados para o nível individual deve ser feita com cautela, evitando inferências causais diretas.

## CONCLUSÃO

Os resultados deste estudo evidenciam uma tendência de queda estatisticamente significativa nas taxas de mortalidade por doenças cerebrovasculares no Brasil ao longo do período analisado, tanto geral quanto estratificada por sexo e regiões, com destaque para as regiões Centro-Oeste e Nordeste, além das faixas etárias mais avançadas.

Por outro lado, observou-se um aumento significativo da mortalidade entre homens jovens (25 a 29 anos), sugerindo a emergência de um novo perfil de risco. Em contraste, as taxas de internação hospitalar mantiveram-se amplamente estáveis em âmbito nacional e regional. No entanto, os custos relacionados às internações por doenças cerebrovasculares apresentaram crescimento significativo em praticamente todas as regiões, faixas etárias e sexos, indicando um aumento do ônus econômico dessas condições, mesmo diante da redução da mortalidade e da estabilidade nas internações.

Esse cenário pode refletir uma maior atenção ao tratamento das doenças cerebrovasculares por parte do sistema de saúde brasileiro, com possível efetividade na redução da mortalidade. Recomenda-se que estudos futuros utilizem dados individuais e incluam variáveis clínicas, sociodemográficas e estruturais. Isso permitirá uma compreensão mais aprofundada dos determinantes econômicos associados ao AVC e poderá subsidiar políticas públicas mais eficazes.

## Data Availability

Os dados estão disponíveis de forma livre e irrestrita através do link: https://datasus.saude.gov.br/

## Footnotes

a Disponível em: https://datasus.saude.gov.br/informacoes-de-saude-tabnet/, por meio de “Informações de Saúde TABNET”, acessando a parte “Epidemiológicas e Morbidade” dentro de “Morbidade Hospitalar do SUS (SIH/SUS)”, para os macrodados de internações hospitalares por doenças cerebrovasculares, e “Estatísticas Vitais” dentro de “Mortalidade desde 1996 pela CID-10”, para os macrodados de óbitos por doenças cerebrovasculares.
